# Leukemoid reaction in sarcomatoid renal cell carcinoma: a two-case report

**DOI:** 10.1186/1477-7819-12-100

**Published:** 2014-04-19

**Authors:** Weiping Huang, Feng Wang, Yeping Li, Feifei Duan, Zhixian Yu

**Affiliations:** 1 Department of Urology, The First Affiliated Hospital of Wenzhou Medical University, Wenzhou 325000, ShangCai Village, Wenzhou, Ou Hai District, China

**Keywords:** Leukemoid reaction, Sarcomatoid, Renal cell carcinoma

## Abstract

Leukemoid reaction is defined as reactive leukocytosis exceeding 40 × 10^9^/l, with a significant increase in early neutrophil precursors, and can be a paraneoplastic manifestation of various malignant tumors. Leukemoid reaction is a sign for poor prognosis in solid tumors so is sarcomatoid renal cell carcinoma (SRCC) when compared to more differentiated histologies. Here, we are reporting two cases of leukemoid reaction after radical nephrectomy, both of which were diagnosed as SRCC pathologically. The operations were successful: no complications were observed and the patients were discharged in good condition. However, a few weeks later, the white blood cell (WBC) count gradually increased. Even though routine management was done immediately, the count was still elevating. A diagnosis of a leukemoid reaction was established and both of them died shortly thereafter. Due to the poor prognosis of most patients with malignant leukemoid reaction, leukemoid reaction may be a predictor of prognosis in patients with SRCC, but more data are needed.

## Background

Leukemoid reaction refers to a reactive leukocytosis, which has been described in response to inflammation, severe or disseminated infection, tissue destruction or other marrow stimulants. Leukemoid reaction is a sign for poor prognosis in solid tumors and so is sarcomatoid renal cell carcinoma (SRCC) when compared to more differentiated histologies. SRCC is a rare but very aggressive variant of renal cell carcinoma (RCC) which used to be thought of as a primary renal sarcoma. Since these tumors co-express both epithelial and stromal markers, they are now believed to represent a form of dedifferentiated carcinoma. As SRCC is highly resistant to chemotherapy and easily metastases, many patients died shortly after diagnosis. Leukemoid reactions have been described mainly in association with lung, gastrointestinal, bladder and head and neck cancers, but have rarely been described in association with SRCC. To our knowledge, these are the first two cases report to do so. The characteristics of both patients are described in Tables 1 and 2.

**Table 1:** Baseline and clinical characteristics (Case 1)

**Characteristics**	
Age (year)	36
Sex	female
BMI (Kg/m^2^)	22.5
Tumor size (cm)	10
TNM stage	
T	T2
N	N1
M	M0
Stage	III
Whole blood count	
Red blood cell	3.43*10^12/L
Hb	102 g/L
Platelet	110*10^9/L
White blood cell	47.2*10^9/L
NEUT%	89.2
Lactate dehydrogenase	1075 U/L
Alkaline phosphatse	434 U/L
Operation	nephrectomy
	free margins with 2/7 positive lymph nodes in posterior peritoneum
Immunohistology	
	CAM5.2(+)CD10(+)CD15(+)CEA(-)CK20(-)CK7(-)RCC(-)VIM(+)
Reason for death	multiple organ failure

**Table 2:** Baseline demographic and clinical characteristics (Case 2)

**Characteristics**	
Age (year)	56
Sex	male
BMI (Kg/m^2^)	24.5
Tumor size (cm)	10
TNM stage	
T	T2
N	N0
M	M0
Stage	II
Whole blood count	
Red blood cell	3.23*10^12/L
Hb	98 g/L
Platelet	258*10^9/L
White blood cell	30.2*10^9/L
NEUT%	91.3
Lactate dehydrogenase	558 U/L
Alkaline phosphatse	289U/L
Operation	nephrectomy
	free margins with 0/5 negative lymph nodes in posterior peritoneum
Immunhistology	
	CAM5.2(+)EMA(-)SMA(+)DES(-)CgA(-)S-100(-)CK20(-)CK7(-)RCC(-)VIM(+)
Reason for death	multiple organ failure

## Case presentation

### Case one

**Figure 1:**
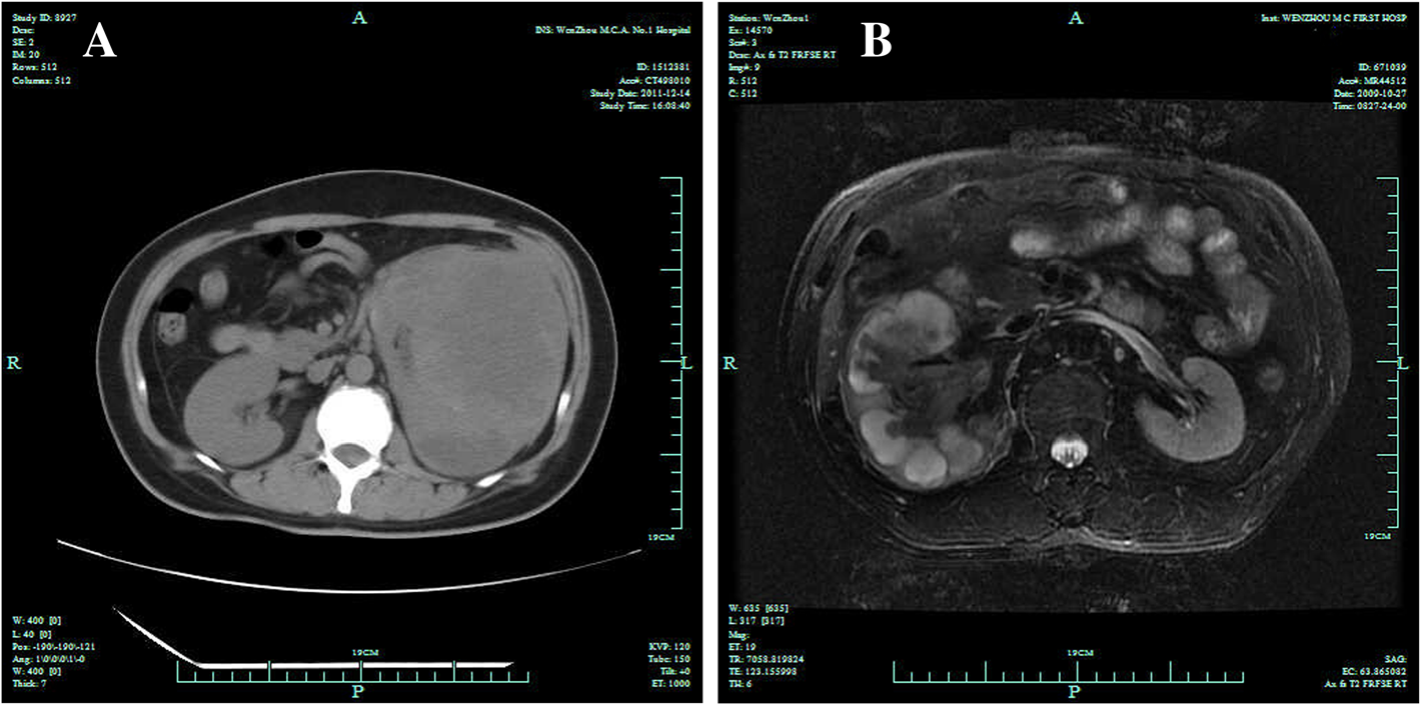
**Scans of the left kidney mass. A**. CT scan: a mass measuring 8 × 10 cm in the left kidney. **B**. MR: A giant abnorminal signal in the right kidney, measuring 9 × 10 cm. Hydronephrosis existed.

**Figure 2:**
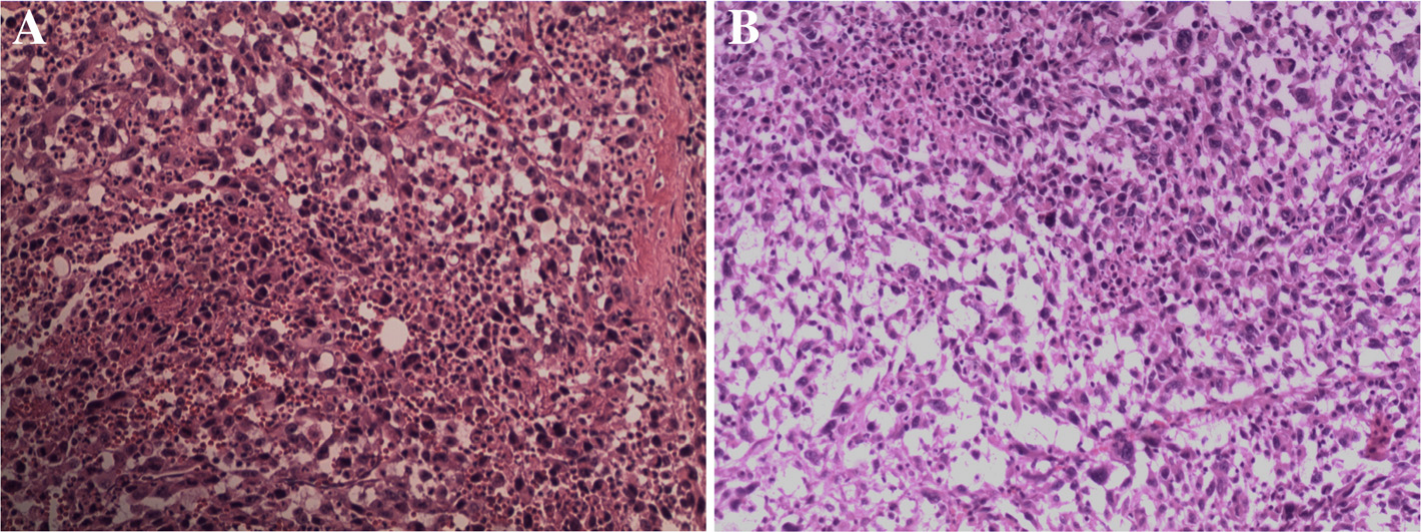
**Pathological examination of sarcomatoid renal cell carcinoma cells. A**. Sarcomatoid renal cell carcinoma was confirmed by the pathological examination. **B**. Sarcomatoid renal cell carcinoma was confirmed by the pathological examination.

**Figure 3:**
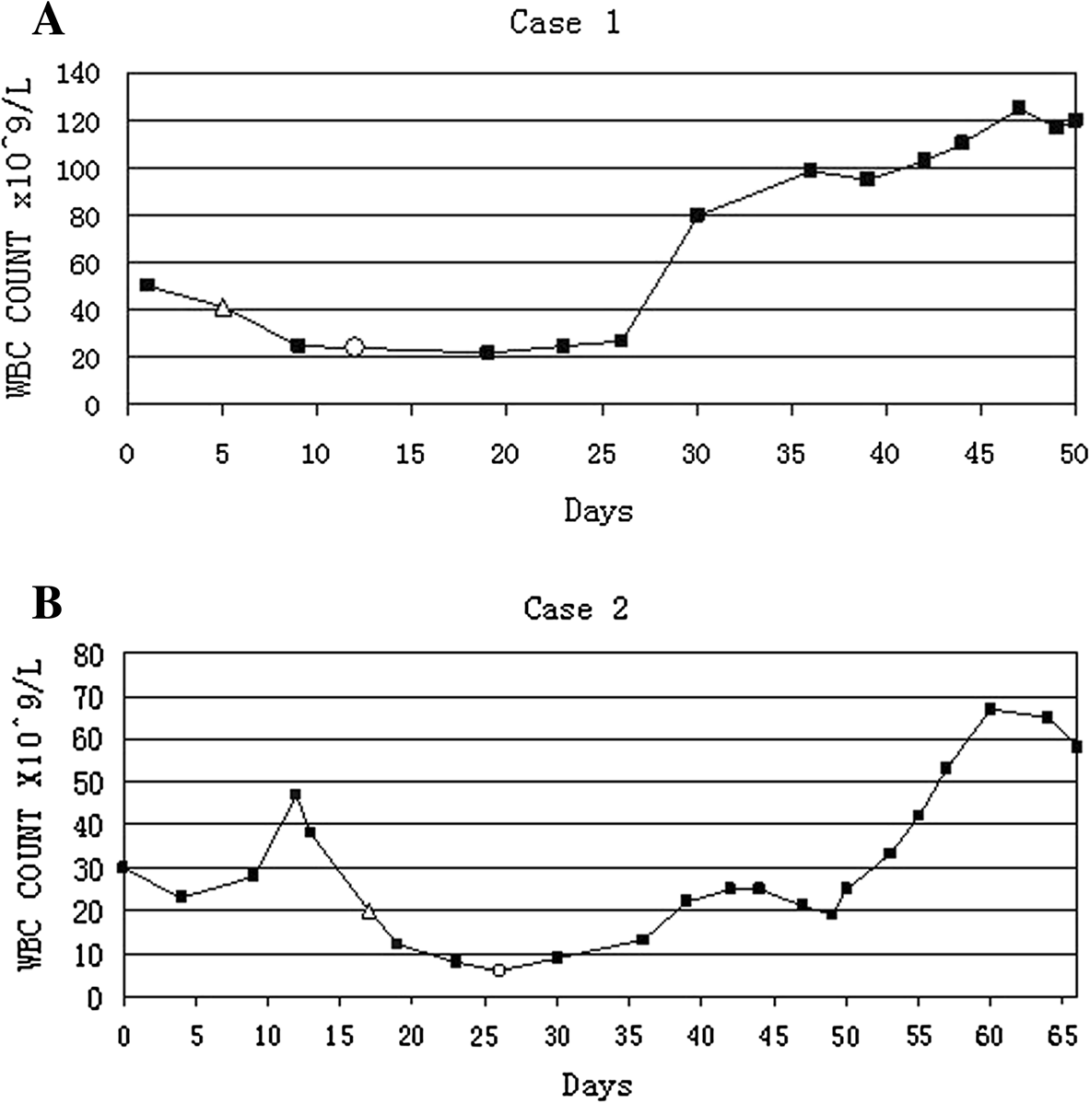
**WBC count graphs showing WBC count was record down when patients were admitted to hospital**. **A**. WBC gradually increased postoperatively. Δ represents the first day patient underwent radical nephrectomy. ○ represents the day patient was discharged from hospital. **B**. WBC count gradually increased postoperatively. Δ represents the first day the patient underwent radical nephrectomy. ○ represents the day the patient was discharged from hospital. L, liter; WBC, white blood count.

A 36 year old woman presented with left flank pain for one month. The patient was admitted to the First Affiliated Hospital of Wenzhou Medical University and underwent serial examinations. Physical examination showed a left flank mass with tenderness. An abdominal computed tomography (CT) scan showed a mass measuring 8 × 10 cm in the left kidney (Figure 1A). Her initial white blood cell (WBC) count was 48 × 10^9^/l, she did not complain of fever, and her bone marrow smear did not show myeloproliferative process. A septic workup, including chest x-ray, blood culture, and urine culture were all negative. She underwent radical nephrectomy and the pathologic diagnosis was SRCC (Figure 2A). No complication developed and the course of treatment was uneventful. However, the WBC count gradually increased (Figure 3A) and the body temperature was high (37.9 to 40.5°C). She was treated with systemic antibiotics and the temperature decreased but the WBC count remained high. Due to the high WBC count, a workup for chronic myeloid leukemia (CML) was initiated. The lactic dehydrogenase (LDH) level was 351. The leukocyte alkaline phosphatase (LAP) score was 280. A peripheral smear showed numerous segmented neutrophils and bands. A bone marrow biopsy also provided no evidence supporting either a hematological neoplasia, or the presence of infiltration by tumor cells. Cytogenetic studies were normal. A PCR(Polymerase Chain Reaction) for BCR-ABL fusion transcript was negative. A diagnosis of a leukemoid reaction was confirmed and she died of multiple organ failure shortly there after at six weeks postoperatively.

### Case 2

The patient, a 56 year old male, complained about more than half a month’s history of right flank pain and fever. He had no systemic and lower urinary tract symptoms and was admitted to the First Affiliated Hospital of Wenzhou Medical University for further examinations. The patient started with a high fever (38.5 to 41.0°C). His first WBC count was 26.9 × 10^9^/L. A tender, palpable right flank mass with an acute percussion pain was detected during a physical examination. His CT scan showed a solid hypo dense lesion in the right kidney measuring 7 × 10 cm (Figure 1B). Meanwhile, a right renal tumor was identified with no evidence of extra-renal extension or distant metastases. An open right radical nephrectomy was performed via a supra-11th rib right flank incision. The tumor was pathologically diagnosed as SRCC (Figure 2B). Postoperative recovery was uncomplicated and white blood cell decreased gradually to the normal range. However, two weeks later after the patient was discharged, he presented with a high fever again once more. Even though broad-spectrum antibiotics were administered without delay, the increasing speed of the WBC count was dramatic (Figure 3B). Leukemoid reaction was established and the patient died of multiple organ failure less than 50 days after the operation.

## Conclusions

Leukemoid reaction is firstly defined by Krumbhaar to describe a phenomenon concerning patients with blood findings that resembling some type of leukemia, but in whom leukemia is not confirmed through the subsequent course of the illness [1]. There is also a more restrictive definition in which the WBC count exceeding 40.000/ul is associated with a cause outside the bone marrow, or a certain proportion of blast cells are present in the blood [2]. Leukemoid reactions are probably caused by mechanical stimuli on bone marrow, resulting from bone metastases. They may also be caused by humoral stimuli resulting from neosynthesized blastic factors, or factors released from the foci of tumor necrosis.

Leukemoid reactions in carcinoma have often been described in association with carcinomas of the stomach, colon, liver, gall bladder, pancreas, lung, bladder, bone, and thyroid gland [3-8]. They have been reported in association with renal cell carcinoma RCC [9], but not with SRCC. To our knowledge, it is the first time that a leukemoid reaction has been reported in association with SRCC.

Both of the two patients had fever during the treatment and observation process. However, fever is an integral component of a leukemoid reaction, often associated with other paraneoplastic syndromes, resulting from either a release of endogenous pyrogens or due to necrotic-inflammatory phenomena of the tumor [10]. Standard antipyretic drugs and antibiotics are usually ineffective. Interleukin I (IL-1) and tumor necrotic factor (alpha) centrally acting on the thermoregulatory center of the hypothalamus are the major endogenous pyrogens [11,12].

A granulocyte colony-stimulating factor (G-CSF) driven reaction is usually seen in advanced local or metastatic disease. It is thought to be associated with tumor progression and necrosis. With the function of this auto–production hematopoietic growth factor, precursor cells in the bone marrow are stimulated to proliferation and maturation into fully differentiated neutrophils [13,14]. Generally, the WBC count will decrease to the normal range if the cause of a leukemoid reaction is eliminated. However, in these two patients, tumor resection did not result in the return of the WBC count. The final WBC count before death was extremely high.

Sarcomatoid differentiation of RCC occurs in all histologic subtypes of RCC, with the incidence ranging from 1.2% to 23.6% [15]. It is uniformly associated with a poor prognosis, with a median survival ranging from 2 to 9 months [15-17]. Surgery, alone, is often inadequate to cure patients, although there is no proven rule for adjuvant therapy. We performed a review of SRCC treatment these 10 years, finding that there was a case report indicating that sunitinib could be helpful for advanced SRCC [18]. Whether the application of tyrosin-kinase inhibitors such as sunitinib would be beneficial for a leukemoid reaction in SRCC is unclear, but worth trying.

These two cases indicate that leukemoid reaction may be a predictor of prognosis in patients with SRCC. Due to most of the patients with a malignant leukemoid reaction having a poor prognosis, it should always keep in mind that the sarcomatoid possibility of the tumor origin when there is an unknown etiology of leukecytosis, especially when infection is not likely. Clearly, more data and evidence are still needed to carry out extensive hematological diagnostic tests in order to distinguish the cause of the leukemoid reaction which would make it easier to find an effective adjuvant therapy.

## Consent

Written informed consent was obtained from these two patient for publication of this two case report and any accompanying images. A copy of the written consent is available for review by the Editor-in-Chief of this journal.

## Abbreviations

SRCC: sarcomatoid renal cell carcinoma; WBC: white blood cell; RCC: renal cell carcinoma; CML: myeloid leukemia; LAP: alkaline phosphatase; PCR: Polymerase Chain Reaction; G-CSF): granulocyte colony-stimulating factor.

## Competing interest

The authors declare that there is no conflict of interest.

## Authors’ contributions

WH: conception and design of the study, acquisition of data, and interpretation of data. WHFW: drafting the manuscript and revising it critically for important content. YL: drafting the manuscript and revising it critically for important content. FD: finished partial manuscript and translation. ZY: conception and coordination of the study. All authors have read and approved the final manuscript.

## Authors’ information

Weiping Huang and Feng Wang: Co-first coauthors.
